# A Moderate Dose of Alcohol Does Not Influence Experience of Social Ostracism in Hazardous Drinkers

**DOI:** 10.3389/fpsyg.2016.00555

**Published:** 2016-04-20

**Authors:** Joseph Buckingham, Abigail Moss, Krisztina Gyure, Neil Ralph, Chandni Hindocha, Will Lawn, H. Valerie Curran, Tom P. Freeman

**Affiliations:** ^1^Department of Biological and Experimental Psychology, Queen Mary University of LondonLondon, UK; ^2^Clinical Psychopharmacology Unit, University College LondonLondon, UK; ^3^Research Department of Clinical, Educational and Health Psychology, University College LondonLondon, UK

**Keywords:** alcohol, social, ostracism, exclusion, Cyberball, fundamental needs, hazardous drinking, age

## Abstract

Anecdotal and correlational evidence suggests a relationship between social ostracism and alcohol dependence. Furthermore, a recent fMRI investigation found differences in the neural correlates associated with ostracism in people with alcohol dependence compared to healthy controls. We predicted that acutely administered alcohol would reduce the negative effects of social ostracism. Alcohol (0.4 g/kg) or matched placebo was administered to a sample of 32 hazardous drinkers over two sessions in a randomized, double-blind, cross-over design. In each session, participants were exposed to an ostracism event via the computerized ball passing game, “Cyberball.” In order to quantify the effects of ostracism, the fundamental needs questionnaire was completed twice on each testing session; immediately after (i) social inclusion and (ii) social exclusion. Ostracism caused robust changes to scores on the fundamental needs questionnaire, in line with previous literature. Alcohol administration did not influence the effects of simulated social ostracism, which was supported by a Bayesian analysis. Exploratory analyses revealed a negative relationship between age and ostracism induced fundamental needs threat across both sessions. In conclusion, a moderate dose of alcohol did not influence experience of simulated social ostracism in hazardous drinkers. Further research is needed to establish the effects of alcohol administration on social ostracism using different doses and populations of alcohol users.

## Introduction

Humans possess an innate need to belong socially and to feel connected to others (Baumeister and Leary, 1995). It follows that social exclusion, also known as ostracism, has long been considered one of the fundamental sources of human anxiety (Horney, 1945). Indeed, research indicates that ostracism has occurred across different cultures and throughout history, as well as in different social animals (see Barner-Barry, 1986). A number of pioneering experiments conducted by Williams and colleagues have shown that social ostracism, simulated via the computerized ball passing paradigm “Cyberball,” results in negative changes to participant self-reports of control, belongingness, self-esteem, and meaningful existence, the “fundamental needs” variables (for review, see Williams, 2007).

Individuals suffering from problematic substance use appear to experience considerable social ostracism. Alcohol-dependent individuals are often ostracized from the general population, as well as by primary caregivers (Pescosolido et al., 2010). It has, therefore, been hypothesized that such exclusion is a contributing factor to the initiation and maintenance of alcohol addiction (Schomerus et al., 2011). It is less clear how or why ostracism might lead to the development of problematic alcohol use, and how it might interact with acute alcohol consumption. Ostracism, by definition, lowers social connectedness, as well as the perception of social connectedness as demonstrated by its effect on the fundamental need *belongingness* (Williams and Sommer, 1997; Williams et al., 2000); defined as an individual’s emotional need to be included in different social groups (Fiske, 2004). Lower social connectedness has a strong connection to negative outcomes in both physical and mental health (see Jetten et al., 2011), including reduced self-efficacy in addiction recovery (Buckingham et al., 2013). Furthermore, an ostracism event has been found to cause increased stress (Zadro et al., 2005), which is also associated with a greater likelihood of both physical and mental health problems (see Thoits, 2012), including drug abuse and relapse (Sinha, 2001). This evidence tentatively suggests that social exclusion may contribute to problematic alcohol use.

To our knowledge, four investigations have more directly addressed the association between ostracism and alcohol use (Maurage et al., 2012; Rabinovitz, 2014; Bacon et al., 2015; Hales et al., 2015). Maurage et al. (2012) examined the neural correlates of social ostracism in abstinent alcohol dependent individuals compared to a non-dependent control group. Participants were exposed to an ostracism event via the Cyberball paradigm (Williams et al., 2000) during functional Magnetic Resonance Imaging (fMRI). Those with alcohol dependence showed increased activation of areas previously linked with experiencing social ostracism, such as the dorsal anterior cingulate cortex and insula (Eisenberger et al., 2003). Moreover, alcohol dependent individuals exhibited less activation in frontal regions (middle frontal cortex and ventrolateral prefrontal cortex), which are thought to inhibit the negative feelings associated with social exclusion (Eisenberger and Lieberman, 2004). This suggests those with alcohol dependence show different neural responses to ostracism, but does not indicate the possible effects of direct alcohol intoxication on responses to ostracism, or the impact an ostracism event could have on alcohol consumption.

Research has also examined whether an ostracism event leads to increased consumption of alcohol. Rabinovitz (2014) conducted a study in which participants were exposed to either ostracism or inclusion, followed by an opportunity to consume a drink they believed contained alcohol. It was found that participants who were ostracized, as opposed to included, were more motivated to drink. In a similar study, Bacon et al. (2015) gave college students a ‘mock taste test’ of beer after inclusion or exclusion on the Cyberball task. Conversely, the authors found that women drank less beer after exclusion than inclusion, whilst no effect was found in men. The evidence is, therefore, unclear as to whether an ostracism event leads to increased alcohol consumption.

An alternative hypothesis that could explain the link between alcohol and ostracism is that alcohol intoxication acts to moderate the negative experience associated with ostracism. This is consistent with research that has shown acute alcohol is capable of decreasing negative affect (Bartholow et al., 2012). Indeed, alleviation of negative affect is thought to be a key mechanism underlying drug addiction (Baker et al., 2004). Consistent with this hypothesis, Hales et al. (2015) proposed that alcohol intoxication may reduce “social pain resulting from ostracism,” in a similar manner to physical pain. This hypothesis was tested in an ecological study conducted in a bar, in which participants were exposed to Cyberball via an IPad. Subjective intoxication predicted both greater distress following exclusion, but also following inclusion, whilst blood alcohol concentration was not significantly related to distress following either inclusion or exclusion. The authors concluded that alcohol intoxication leads to reduced pain from exclusion, but also reduced pleasure from inclusion. However, it is difficult to establish causality using cross-sectional designs, thus, a controlled laboratory study that manipulated alcohol, as well as ostracism, would provide further evidence to support this finding.

In order to further investigate this, we used a placebo-controlled crossover design to establish the acute effects of alcohol on the experience of ostracism in drinkers who are at risk of escalating into problematic use. We predicted, first, that ostracism would cause changes to the fundamental needs variables, consistent with previous research (see Williams, 2007). Second, on the basis of previous findings examining alcohol intoxicated participants (Hales et al., 2015), we predicted that acute alcohol consumption would reduce the negative impact social ostracism on the fundamental needs variables.

## Materials and Methods

### Participants and Design

This study was approved by the UCL Ethics Committee, and all participants gave written informed consent. The sample consisted of 32 volunteers (16 women) recruited on an opportunity basis from the local community. Each participant received an acute dose of both alcohol and placebo across two sessions in a randomized, balanced, and double-blinded crossover design. A moderate dose (0.4 g/kg; Holdstock and de Wit, 1998) was chosen for this initial experiment since high doses (e.g., 0.8 g/kg) can cause global impairment (Bisby et al., 2010) which might have interfered with participants’ ability to understand and engage with the Cyberball task. Based on a sample size of 32, α = 0.05, β = 0.8, 4 measurements, and a correlation between measures of 0.5, a power calculation conducted in G^∗^Power ^∗^(3.1.9) suggests this design should be adequately powered to detect a medium effect of alcohol administration on ostracism-inducted fundamental need threat (*f* = 0.21).

Inclusion criteria were as follows: age 18–40, reporting alcohol use at least once per month, scoring “hazardous” (a mark of 8 or above) on the Alcohol Use Disorders Identification Test (AUDIT; Babor et al., 2001). We chose to recruit a sample of hazardous drinkers, as they reflect a group who are ‘healthy’ and non-treatment seeking, but *at risk* of developing an alcohol use disorder. Participants were excluded if they reported a diagnosis of dependence on alcohol or any other drug in their lifetime (due to the ethical considerations of alcohol administration), other than nicotine. Due to the focus on alcohol, further data on other drug use was not collected. No participants reported any psychiatric illness. Participants were asked to abstain from using any drugs other than nicotine for at least 24 h before each testing session.

### Alcohol Administration and Blinding

Either alcohol or matched placebo was administered to participants using an established protocol (Bisby et al., 2010). An assistant prepared the alcoholic or matched placebo drinks, ensuring that both the experimenter and participant remained blind to study condition. Alcoholic drinks consisted of ethanol (90% vol/wt) diluted with tonic water (Schweppes, Uxbridge, UK), and were split equally between ten 50 ml cups. In order to disguise the taste, two drops of Tabasco hot sauce (McIhenny, Avery Island, LA) were added to each of the ten beverages. The placebo drinks consisted of ten 50 ml portions of tonic water, also containing two drops of Tabasco hot sauce.

### Assessments

#### Cyberball (Williams et al., 2000)

**FIGURE 1:**
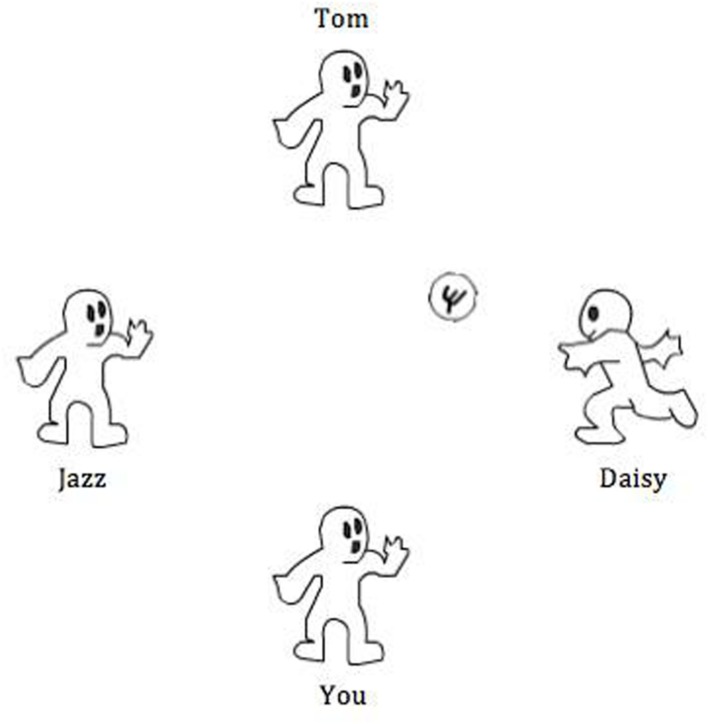
**An example of the screen viewed by participants during Cyberball**.

Participants were instructed that they would be taking part in a mental visualization experiment, and that “conceptualization of the game” was the purpose of the task. Participants were also told that in-game performance did not matter to the study. After entering their name, participants began a computerized ball passing game involving three other players (**Figure 1**). When the ball was received, the participant clicked on one of the three other player’s avatars to pass the ball on. In reality, the other players were computer controlled. Each of the three avatars had a nametag alongside it. In order to control for any possible effects of gender, the avatar names consisted of one male, one female, and one gender ambiguous name. Participants were exposed to a different set of names during each session of Cyberball, which were counterbalanced across testing sessions. Each session of Cyberball lasted approximately 3 min.

#### Ostracism Manipulation Check (Gonsalkorale and Williams, 2007)

In order to assess participant perception of the manipulation, after each game they reported the percentage of throws they believed they had received. They also completed a 5-point, Likert-type scale ranging from “Not at all” (1) to “Very much” at (5) entitled: “I was ignored and I was excluded.”

#### The fundamental Needs Variables (Gonsalkorale and Williams, 2007)

A self-report questionnaire designed to measure the following need constructs: control, self-esteem, belonging, and meaningful existence. This scale was developed specifically for use with the Cyberball task and has demonstrated sensitivity to simulated social ostracism in a number of studies (Hartgerink et al., 2015). Each construct was assessed via three statements. Participant agreement with each statement was recorded on a 5-point, Likert-type scale ranging from “Not at all” at 1 to “Very much” at 5. Following Gonsalkorale and Williams (2007) each construct was scored from 1 to 5, with higher scores representing greater need satisfaction. Composite scores (combinations of the four constructs, again scored 1–5) were used as the primary outcome variable in this study as we did not have specific hypotheses about individual constructs.

#### Alcohol Use Disorder Identification Test (AUDIT; Babor et al., 2001)

A 10 item scale designed for screening problematic alcohol use. Scores range from 0 to 40, with higher scores indicating increasingly problematic use, expressed as ‘hazardous drinking’, ‘harmful drinking,’ and ‘dependence.’

### Procedure

Each testing session began with a Breathalyzer test (Lion alcometer 500) in order to ensure the participant had not consumed alcohol. Next they were instructed to consume each of the ten alcohol/placebo beverages at 3-min intervals (over 30 min in total). A breathalyzer test was administered 10 min after the last drink had been consumed to allow time for absorption. Participants then completed a visual probe and picture rating task which will be reported elsewhere (see Freeman et al., 2015 for details of the tasks). Next, the Cyberball paradigm was administered to participants in order to simulate social ostracism. All participants completed two Cyberball games during each testing session in a fixed order (inclusion first, exclusion second) to avoid carry-over effects of social ostracism (see Eisenberger et al., 2003). In the inclusion session, they were equally included, receiving 25% of 60 ball passes throughout the game. In the second (exclusion) game, they initially received two passes at the beginning of play. After that, they are then excluded for the remainder of the game. Manipulation check questions and the fundamental needs variables were completed immediately after each game. After this, a third Breathalyzer reading was taken. Finally, at the end of each testing session, participants were asked which drink they received (alcohol or placebo). Each testing session was conducted 1 week apart. Participants were debriefed about the study aims at the end of the second testing session.

### Statistical Analyses

The fundamental needs variables and ostracism manipulation checks were analyzed by means of 2 × 2 repeated measures ANOVA with the within subjects factors of ostracism (inclusion/exclusion) and alcohol (placebo/alcohol). Since this study used a crossover design (*n* = 32) balanced for gender, additional exploratory analyses were carried out with (i) gender and (ii) order of alcohol administration, as additional between-subject factors. Exploratory correlational analyses were also carried out between subjective response to ostracism, levels of alcohol use, and age. Guesses on treatment were analyzed using χ^2^ tests. All *post hoc t*-tests were corrected locally using the Bonferroni method. Pearson correlational analyses are presented using uncorrected *p* values. Degrees of freedom and *p* values were corrected using the Greenhouse–Geisser technique where appropriate. The following missing data (%) were imputed with the group mean: percentage of throws on the Cyberball task (3.91%), feelings of exclusion after the Cyberball task (0.78%), fundamental needs questionnaire scores (0.39%), and units of alcohol consumed per week (3.13%). In order to evaluate evidence for the null hypothesis, Scaled Jeffreys-Zellner-Siow (JZS) Bayes Factor was calculated. We used a scaled-information prior of *r* = 1, which is the default value recommended by Rouder et al. (2009).

## Results

### Participants, Drinking Behavior and Acute Effects of Alcohol

The mean (SD) age of the sample was 22.53 (4.28), AUDIT scores were 14.75 (5.86) and the number of alcohol units consumed a week was 24.05 (24.54). Blood alcohol concentrations (grams/litres BAC) were 0.00 at the start of every testing session, and increased to 0.50 (0.22) 10 min after drink administration, and 0.42 (0.10) at the end of testing, 35 min after drink administration.

On the placebo session, correct/incorrect treatment guesses were higher than chance for participant (27/5, χ^2^(1) = 8.000, and *p* = 0.005) and experimenter ratings (24/8, χ^2^(1) = 15.125, and *p* < 0.001). On the alcohol session, participant ratings were better than chance (28/4, χ^2^(1) = 18.000, and *p* < 0.001) but experimenter ratings were not (18/14, χ^2^(1) = 0.500, and *p* = 0.480).

### Ostracism Manipulation Checks (**Table 1**)

**Table 1:** Scores from the Cyberball task before and after simulated social ostracism, following administration of placebo and alcohol.

	Inclusion		Ostracism		Alcohol administration by ostracism interaction ($\eta_{p}^{2}$)
	Placebo	Alcohol	Placebo	Alcohol	
**Manipulation check**					
Excluded/ignored	1.69 (0.82)	1.55 (0.80)	3.81 (1.00)	3.75 (0.84)	0.000
% Throws	25.27 (8.91)	24.41 (6.06)	9.03 (3.29)	8.17 (4.00)	0.003
**Fundamental needs**					
Composite score	3.61 (0.54)	3.67 (0.46)	2.52 (0.47)	2.58 (0.53)	0.000
Belonging	4.40 (0.81)	4.42 (0.78)	2.67 (0.92)	2.89 (0.97)	0.032
Self-esteem	3.28 (0.78)	3.23 (0.65)	2.50 (0.71)	2.47 (0.60)	0.000
Control	2.16 (0.97)	2.44 (0.99)	1.48 (0.73)	1.50 (0.56)	0.068
Meaningful existence	4.63 (0.64)	4.58 (0.60)	3.43 (0.93)	3.48 (0.93)	0.005

#### Percentage of Throws Received

Participants perceived a lower number of throws during Cyberball in the exclusion game session compared to the inclusion game session *F*(1,31) = 338.254, *p* < 0.001, and $\eta_{p}^{2}$ = 0.912, reflecting a successful manipulation check for the ostracism procedure. The interaction between alcohol and ostracism [*F*(1,31) = 0.000, *p* = 0.997, and $\eta_{p}^{2}$ = 0.000] and main effect of alcohol [*F*(1,31) = 0.558, *p* = 0.449, and $\eta_{p}^{2}$ = 0.019] were non-significant, suggesting that alcohol administration did not influence participant’s perception of the number of throws received.

#### Experience of Being Ignored and Excluded

There was no significant interaction between alcohol and ostracism [*F*(1,31) = 0.092, *p* = 0.763, and $\eta_{p}^{2}$ = 0.003]. There was a significant main effect of ostracism [*F*(1,31) = 193.663, *p* < 0.001, and $\eta_{p}^{2}$ = 0.862] showing that participants were subjectively aware of being excluded during the task. There was no main effect of alcohol [*F*(1,31) = 0.354, *p* = 0.556, and $\eta_{p}^{2}$ = 0.011], suggesting that alcohol administration did not impair participant’s perception of inclusion or exclusion during the game.

### Effects of Simulated Social Ostracism on the Fundamental Needs Variables (**Table 1**)

Analysis of composite fundamental needs scores revealed a main effect of ostracism [*F*(1,31) = 119.607, *p* < 0.001, and $\eta_{p}^{2}$ = 0.794], no evidence for an effect of alcohol [*F*(1,31) = 0.467, *p* = 0.500, and $\eta_{p}^{2}$ = 0.015], or the predicted alcohol by ostracism interaction [*F*(1,31) = 0.011, *p* = 0.919, and $\eta_{p}^{2}$ = 0.000]. The same pattern of results was found when analyzing each individual construct (all *p*’s > 0.05), providing no evidence for an alcohol by ostracism interaction (**Table 1**).

### Examining Evidence for the Null Hypothesis

A Bayesian analysis was used to examine whether the effects of ostracism on fundamental needs scores differed after alcohol or placebo [the predicted alcohol by ostracism interaction; *F*(1,31) = 0.011, *p* = 0.919, and $\eta_{p}^{2}$ = 0.000]. This analysis indicated that the null is 7.26 times more likely than the alternative given the data, supporting a lack of acute alcohol effects on subjective response to social ostracism (JZS Bayes Factor = 7.26).

### Exploratory Analyses: Gender, Alcohol Use and Age

#### Gender

A main effect of ostracism was found [*F*(1,30) = 119.869, *p* < 0.001, and $\eta_{p}^{2}$ = 0.800]. Interactions between alcohol, ostracism, and gender [*F*(1,30) = 0.182, *p* = 0.673, and $\eta_{p}^{2}$ = 0.006], alcohol and ostracism [*F*(1,30) = 0.010, *p* = 0.920, and $\eta_{p}^{2}$ = 0.000], ostracism and gender [*F*(1,30) = 1.068, *p* = 0.310, and $\eta_{p}^{2}$ = 0.034], alcohol and gender [*F*(1,30) = 0.065, *p* = 0.801, and $\eta_{p}^{2}$ = 0.002], and the main effect of alcohol [*F*(1,30) = 0.453, *p* = 0.506, and $\eta_{p}^{2}$ = 0.015], were all non-significant.

#### Order of Alcohol Administration (**Figure 2**)

**FIGURE 2:**
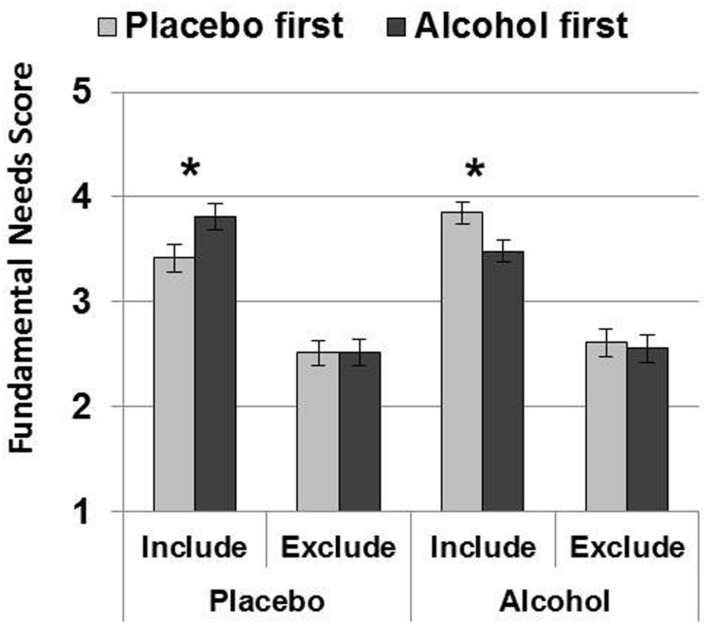
**Order effects analysis**. Mean (SE) Fundamental Needs scores were lower after inclusion on placebo (when placebo was administered first), and lower after inclusion on alcohol (when alcohol was administered first). Thus, scores were always lower on the first testing session, but for inclusion only. Order effects did not influence fundamental need scores after exclusion, and the overall magnitude of ostracism on fundamental need scores were similar after alcohol and placebo. (^∗^*p* < 0.05).

An alcohol by ostracism by order interaction was found [*F*(1,30) = 9.953, *p* = 0.004, and $\eta_{p}^{2}$ = 0.249] as well as an alcohol by order interaction [*F*(1,30) = 6.730, *p* = 0.015, and $\eta_{p}^{2}$ = 0.183] and a main effect of ostracism [*F*(1,30) = 115.892, *p* < 0.001, and $\eta_{p}^{2}$ = 0.794]. This three-way interaction was reflected by differences after inclusion only: lower fundamental need scores were reported on placebo when placebo was administered first (*p* = 0.037 and $\eta_{p}^{2}$ = 0.137) and also on alcohol when alcohol was administered first (*p* = 0.022 and $\eta_{p}^{2}$ = 0.163). By contrast, scores did not differ according to order after exclusion on placebo (*p* = 0.976 and $\eta_{p}^{2}$ = 0.000) or alcohol (*p* = 0.786 and $\eta_{p}^{2}$ = 0.002). The alcohol by ostracism [*F*(1,30) = 0.014, *p* = 0.908, and $\eta_{p}^{2}$ = 0.000], and ostracism by order [*F*(1,30) = 0.037, *p* = 0.848 and $\eta_{p}^{2}$ = 0.001] interactions were non-significant, as was the main effect of alcohol [*F*(1,30) = 0.553, *p* = 0.463 and $\eta_{p}^{2}$ = 0.018]. Thus, neither gender nor order of alcohol administration influenced the effects of alcohol on fundamental needs scores following social ostracism.

#### Alcohol Use

Given the absence of an alcohol by ostracism interaction, fundamental need changes scores (inclusion – exclusion) were collapsed from both placebo and alcohol sessions. These scores were investigated for possible correlations with AUDIT scores and weekly alcohol consumption. No relationship was found between fundamental need change scores and AUDIT scores (*r* = 0.150 and *p* = 0.414), or units of alcohol consumed per week (*r* = 0.236 and *p* = 0.193).

#### Age (**Figure 3**)

**FIGURE 3:**
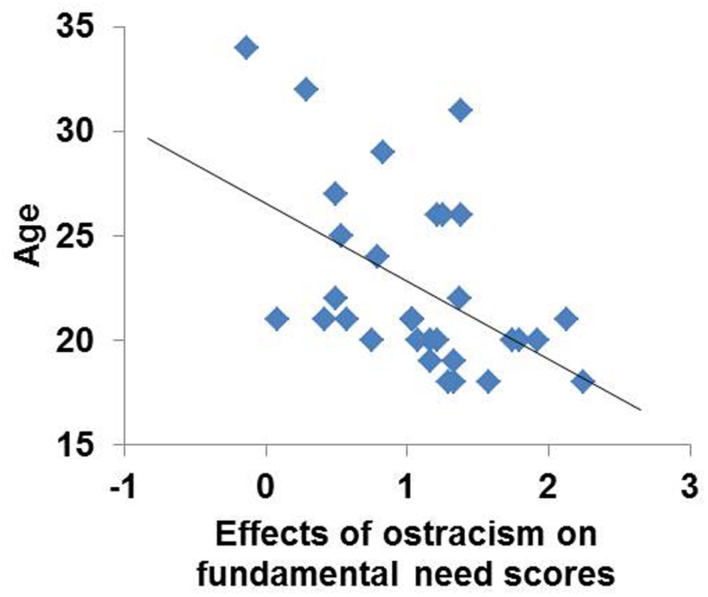
**Effects of simulated social ostracism on fundamental need scores were negatively correlated with age, accounting for 24% of the variance**.

There was a significant negative correlation between fundamental need changes scores and age (**Figure 3**: *r* = 0.493 and *p* = 0.004), indicating that younger participants experienced a greater subjective response to the ostracism manipulation.

## Discussion

This investigation aimed to examine the effects of acute alcohol administration on the response to simulated social ostracism in hazardous alcohol drinkers. As predicted, ostracism caused marked changes to the fundamental needs variables. This is in line with the many experiments that have employed Cyberball as an ostracism manipulation (see Williams, 2007). Contrary to our predictions, however, this effect was not moderated by acute alcohol administration. This null finding was further supported by a Bayesian analysis. We also established that these results were not attributable to effects of gender or order of alcohol administration. As a result, our findings suggest that a moderate dose of alcohol (0.4 g/kg) does not influence the effects of simulated ostracism in hazardous drinkers.

Interestingly, the findings of this investigation contrast with those of Hales et al. (2015), who showed that alcohol successfully moderates the negative response to ostracism. However, whilst they showed that subjective intoxication was related to decreased distress following ostracism, they did not find a relationship when examining objective intoxication (blood alcohol levels). It may be the case, therefore, that subjective intoxication, but not objective intoxication, acts to moderate the negative experience associated with ostracism. Our study also differed because it was conducted in a controlled laboratory environment, as opposed to an ecological setting (a real-world bar). Previous evidence suggests that drinking alcohol in social contexts produces greater feelings of intoxication compared to drinking in non-social contexts (Kirkpatrick and de Wit, 2013). It may be the case, therefore, that the social environment interacts with alcohol intoxication to moderate the response to ostracism, although more evidence is needed to substantiate this claim.

A potential explanation for the null finding of this study concerns alcohol administration. The moderate dose of alcohol did produce clear effects as evidenced by elevated blood alcohol concentrations, and participants’ ability to correctly guess that they had received alcohol. On the other hand, this dose may have been too low to elicit an effect on the experience of ostracism. An advantage of using a moderate dose in this study was the preservation of cognitive functioning, as higher doses can cause global impairment (Bisby et al., 2010). Nevertheless, it is possible that higher and/or repeated doses of alcohol might influence hazardous drinkers’ responses to simulated social ostracism. Given that alcohol dependent patients showed decreases in prefrontal cortical activation during exclusion on Cyberball (Maurage et al., 2012), it stands to reason that a higher dose of alcohol might have effects through greater impairment of executive functioning.

Another consideration is that we recruited hazardous drinkers, with a much lower level of alcohol use problems than dependent drinkers. Perhaps prolonged, heavy alcohol consumption changes the experience of an ostracism event. There is evidence for this claim, as Maurage et al. (2012) found different neural correlates of social exclusion in alcohol dependent individuals compared to healthy controls. As Hales et al. (2015) did not report data concerning drinking behavior, it is difficult to establish whether the participants who took part in their investigation were either normal, hazardous or dependent alcohol drinkers. It may be, therefore, that the severity of alcohol use problems moderates the acute effects of alcohol on experience of social ostracism. This is a possibility that warrants further investigation.

It is also possible that alcohol did influence participants’ response to social ostracism in this study, but at a neural level, that could not be detected using the behavioral methods we employed. For example, Maurage et al. (2012) found that healthy control and alcohol-dependent groups showed the same behavioral response to ostracism but differed according to fMRI measures, which may be more sensitive. On the basis of our data we cannot rule out this possibility, and future studies could benefit from behavioral measures in combination with imaging techniques, as employed by Maurage et al. (2012) and others (e.g., Eisenberger et al., 2003; Eisenberger and Lieberman, 2004). However, previous research has demonstrated drug effects on the Cyberball paradigm using behavioral measures alone (Frye et al., 2014; Hales et al., 2015). Additionally, the negative correlation we found between age and subjective response to ostracism is consistent with previous Cyberball research using behavioral methods (e.g., Sebastian et al., 2010; Hawkley et al., 2011; Pharo et al., 2011) which supports the validity and sensitivity of the experimental methods employed.

A methodological issue with this study concerns unblinding of the placebo/alcohol administration procedure. Participants were able to correctly guess as to whether they had received alcohol or placebo significantly better than chance. It could be, therefore, that either alcohol, or the expectancies associated with alcohol due to a placebo effect, influenced the findings of this study. Given this, it appears that the selected placebo administration procedure (Bisby et al., 2010) may not be appropriate for samples of hazardous drinkers, who may be more sensitive at detecting placebo beverages than non-hazardous drinkers.

## Conclusion

In conclusion, alcohol (0.4 g/kg) did not influence the subjective experience of simulated social ostracism in hazardous drinkers in a controlled laboratory experiment. Future studies should aim to extend our findings by testing higher acute doses, additional (e.g., harmful and dependent) groups of alcohol users, and supplementary techniques such as fMRI.

## Author Contributions

JB conceived the experiment. JB, TF, and HVC designed the protocol. CH and NR assisted with the Cyberball task. HVC wrote the blinding code. WL used the code to prepare drinks. JB, AM, and KG conducted the testing. JB and TF conducted the statistical analysis. JB wrote the first draft of the manuscript. All authors have contributed to and approved the final manuscript.

## Conflict of Interest Statement

The authors declare that the research was conducted in the absence of any commercial or financial relationships that could be construed as a potential conflict of interest.
